# Characterization of Nano- and Microstructures of Native Potato Starch as Affected by Physical, Chemical, and Biological Treatments

**DOI:** 10.3390/foods13132001

**Published:** 2024-06-25

**Authors:** Antonieta Mojo-Quisani, Katiuska Licona-Pacco, David Choque-Quispe, Miriam Calla-Florez, Carlos A. Ligarda-Samanez, Augusto Pumacahua-Ramos, Víctor J. Huamaní-Meléndez

**Affiliations:** 1 Agroindustrial Engineering, National University of San Antonio Abad del Cusco, Cusco 08000, Peru; 134720@unsaac.edu.pe (K.L.-P.); miriam.calla@unsaac.edu.pe (M.C.-F.); 2 Agroindustrial Engineering, José María Arguedas National University, Andahuaylas 03701, Peru; caligarda@unajma.edu.pe; 3 Department of Food Engineering, Universidad Nacional Intercultural de Quillabamba, Cusco 08741, Peru; augusto.pumacahua@uniq.edu.pe; 4 Department of Food Engineering and Technology, São Paulo State University (UNESP), Campus of São José do Rio Preto, São Paulo 15385-000, Brazil; victor.melendez@unesp.br

**Keywords:** acid treatment, enzymatic treatment, ethanolic precipitation, modified starch, thermal, structural, physicochemical characterization

## Abstract

Modifying starch allows for improvements in its properties to enable improved uses in food matrices, bioplastics, and encapsulating agents. In this research, four varieties of native potato starch were modified by acid treatment, enzymatic treatment, and ethanol precipitation, and their physicochemical, structural, thermal, and techno-functional characteristics were analyzed. According to FT-IR analysis, no influence of the modified starches on the chemical groups was observed, and by scanning electron microscopy (SEM), spherical and oval shapes were observed in the acid and enzymatic treatments, with particle sizes between 27 and 36 μm. In particular, the ethanolic precipitation treatment yielded a different morphology with a particle size between 10.9 and 476.3 nm, resulting in a significant decrease in gelatinization temperature (DSC) and more pronounced crystallites (XRD). On the other hand, the enzymatic treatment showed higher values for z-potential (ζ), and the acid treatment showed lower mass loss (TGA). Acid and ethanolic treatments affected the dough properties compared to native starches. The techno-functional properties showed a decrease in the water absorption index, an increase in the water solubility index, and varied swelling power behaviors. In conclusion, the modification of potato starches through acid, enzymatic, and ethanolic precipitation treatments alters their physicochemical properties, such as swelling capacity, viscosity, and thermal stability. This in turn affects their molecular structure, modifying morphology and the ability to form gels, which expands their applications in the food industry to improve textures, stabilize emulsions, and thicken products. Furthermore, these modifications also open new opportunities for the development of bioplastics by improving the biodegradability and mechanical properties of starch-based plastic materials.

## 1. Introduction

Starch, a polysaccharide abundant in nature, is positioned as a renewable biomaterial with low environmental impact [1]. Ranked as the second most abundant after cellulose, this natural component finds applications in various sectors [2]. Its characteristics as a thickener, adhesive, and gelling agent make it a key ingredient in the food and textile industries [3]. Among these properties, its ability to form thermoreversible gels stands out, which makes it ideal for the encapsulation and controlled release of bioactive compounds in pharmaceutical and food applications. In addition, its ability to interact with other materials, such as proteins and lipids, broadens its applications in the food industry, improving the stability of emulsions and foams. The filmogenic properties of starch make it an ideal material for the creation of edible films and biodegradable coatings, which is beneficial in food applications.

However, native starch has limitations that restrict its potential applications [4]. To overcome the limitations of native starch, its modification is performed through chemical, physical, and enzymatic methods [3]. These techniques allow for the adjustment of starch properties, such as particle size, stress resistance, thermal properties, and retrogradation behavior [5].

The amylose and amylopectin contents in starch are crucial when starch is subjected to chemical, biological, or physical modifications due to their impact on the final properties of the modified starch. Amylose, being linear (more ordered and crystalline structures), tends to form stronger gels with higher retrogradability [6], which can influence the texture and stability of food products. On the other hand, amylopectin, being branched (less crystalline structures), contributes to the formation of more flexible gels and is less prone to retrogradation. When modifications are made to starch, the relative contents of amylose and amylopectin can affect the swelling capacity, solubility [7], crystallinity, granule size, chemical nature, and arrangement of polymers within the granule [8]. Therefore, understanding and knowing the proportions of amylose and amylopectin are essential to adjusting the properties of modified starch according to the specific needs of different applications. 

Chemical modification introduces functional groups into the starch molecule, resulting in highly functional derivatives with improved properties [9]. These modifications can technically improve aspects such as morphology, crystalline structure, thermal behavior, and even particle size reduction [4,10].

Its high solubility, thermal stability, non-toxicity, and low cost have led to the use of nanostarch in numerous applications, including drug delivery, cosmetics, textiles, food, enhanced oil recovery (EOR), and tissue engineering [9,11,12]. Of particular note is the effectiveness of nanostarch in inducing the stabilization of Pickering emulsions without the need for surfactants [9]. These applications take advantage of the special functions that can be achieved by modifications in the structure and properties of native starch [13].

Acid hydrolysis, a commonly used method to obtain nanostarch with high crystallinity and stability, is often combined with other techniques such as ultrasound, nanoprecipitation, milling, and crosslinking to optimize starch properties [14,15]. The efficiency depends on several factors during the hydrolysis process, such as the type and concentration of acid used, the reaction time and temperature, and the source of the starch itself [16].

Enzymatic modification offers another approach that directly affects starch properties by creating a new structure [13]. Factors such as enzyme concentration, incubation times, enzyme mixture, reaction temperature, and pH can significantly influence the degree of hydrolysis and various functional properties [15,17]. These factors can also influence water-holding capacity and stickiness, paving the way for “clean-label” starches with unique gelling properties [18]. Enzymatic processing, combined with techniques such as nanoprecipitation, high-pressure homogenization, and ultrasonication [2,19], finds applications in the production of Pickering emulsions, films, and nanocomposites [2,20]. In addition, modified starches exhibit reinforcing properties in biocomposites, making them valuable not only for their functionality but also for their renewable and biodegradable nature [1].

Recent research has explored the antisolvent precipitation method to produce biopolymeric nanoparticles from native starches [21,22]. This rapid method typically uses sonication, gamma irradiation, mild combinations of alkaline hydrolysis, and antisolvents, such as acetone or ethanol, added to the starch solution [14,23,24,25]. Some studies suggest that the dropwise addition of ethanol to a weak solution of gelatinized starch while stirring promotes the formation of smaller nanoparticles [26].

In short, starch, with its exceptional properties and the possibility of obtaining nanoparticles and/or microparticles with even more versatile characteristics, is positioned as a biopolymer with great potential for the development of new materials and technologies in various sectors. In particular, biopolymeric nanoparticles often exhibit higher solubility and stability in water compared to their unmodified counterparts [21].

This study aims to produce nanostructures and microstructures of native potato starches modified by acidic, enzymatic, and ethanolic precipitation treatments to characterize their physicochemical, structural, and thermal properties, which will allow us to evaluate their potential use in various food applications.

## 2. Materials and Methods

### 2.1. Materials and Reagents

Four native potato varieties, cultivated in the Cusco region of Peru, were utilized in the investigation: two sweet varieties, Aq’hu Pukucho (APE) (S: 14°44′36.5″; W: 71°26′22.9″; altitude 3925 m) and Yurakk Kkachun Wakkachi (YKW) (S: 14°05′19.8″; W: 71°17′55.8″; altitude 3475 m), and two bitter varieties, Yurac Anca (YA) (S: 14°52′19.8″; W: 71°31′48.4″; altitude 4001 m) and Huarmi Mallco (HM) (S: 14°05′19.8″; W: 71°17′55.8″; altitude 3475 m). These varieties may exhibit variations in starch composition, potentially influencing their behavior during modification. Starch extraction was conducted via the wet method, with the resulting samples utilized for various modification treatments. Reagents included α-amylase enzyme (Amylase Liquid IFCC, Quimica Clinica Aplicada S.A., Amposta, Spain), orthophosphoric acid with a minimum purity of 85% (Merck KGaA, Darmstadt, Germany, CAS 7664-38-2), and ethanol with 96% purity (Alkofarma, Lima, Peru). All solutions were prepared using distilled water.

### 2.2. Acid Treatment

The method outlined by Ahmad et al. [23] was employed with certain adaptations. Five grams of starch were dispersed in 50 mL of orthophosphoric acid (1 M). The solution underwent ultrasonication at 40 Hz (Biobase equipment, model UC-08 Myanmar, Silicon Valley, CA, USA) for 30 min at 40 °C. Subsequently, the supernatant was separated via centrifugation at 10,000× *g* for 10 min, and the resulting sediment was washed eight times with distilled water under continuous stirring until reaching a neutral pH. The resultant product was vacuum dried (10 mBar) at 20 °C for 4 h using a vacuum oven (BINDER, model VD56, Tuttingen, Germany), followed by sieving through a UTEST electric sieve (model UTG-0416, Lima, Peru) with a mesh size of 63 μm. Finally, the samples were packaged for subsequent characterization.

### 2.3. Enzymatic Treatment

This procedure was conducted following the protocol proposed by Dukare et al. [1] with certain modifications. A solution was prepared containing 1 g of starch and 100 mL of distilled water. Subsequently, 50 µL of α-amylase enzyme (A = buffer/enzyme and B = buffer/substrate) was added and mixed using a magnetic stirrer (CAT, model M6, Staufen, Germany). The reaction temperature was maintained at 50 °C using an incubator (BINDER, model DIN 12880, Tuttlingen, Germany) for 4 h with continuous stirring (BINDER, model VD56, Tuttingen, Germany). The resulting sample was cooled to room temperature and centrifuged (Centurión, 1.C2024, Chichester, UK) at 4000 rpm for 10 min to separate the supernatant. The obtained samples were then vacuum dried (10 mBar) (BINDER, model VD56, Tuttingen, Germany) at 20 °C for 4 h and ground using a Retsch PM100 planetary ball mill (Haan, Germany). Finally, the material was sieved through a 63 μm mesh and packed for the evaluation of its physicochemical and structural properties.

### 2.4. Ethanolic Precipitation

**Figure 1:**
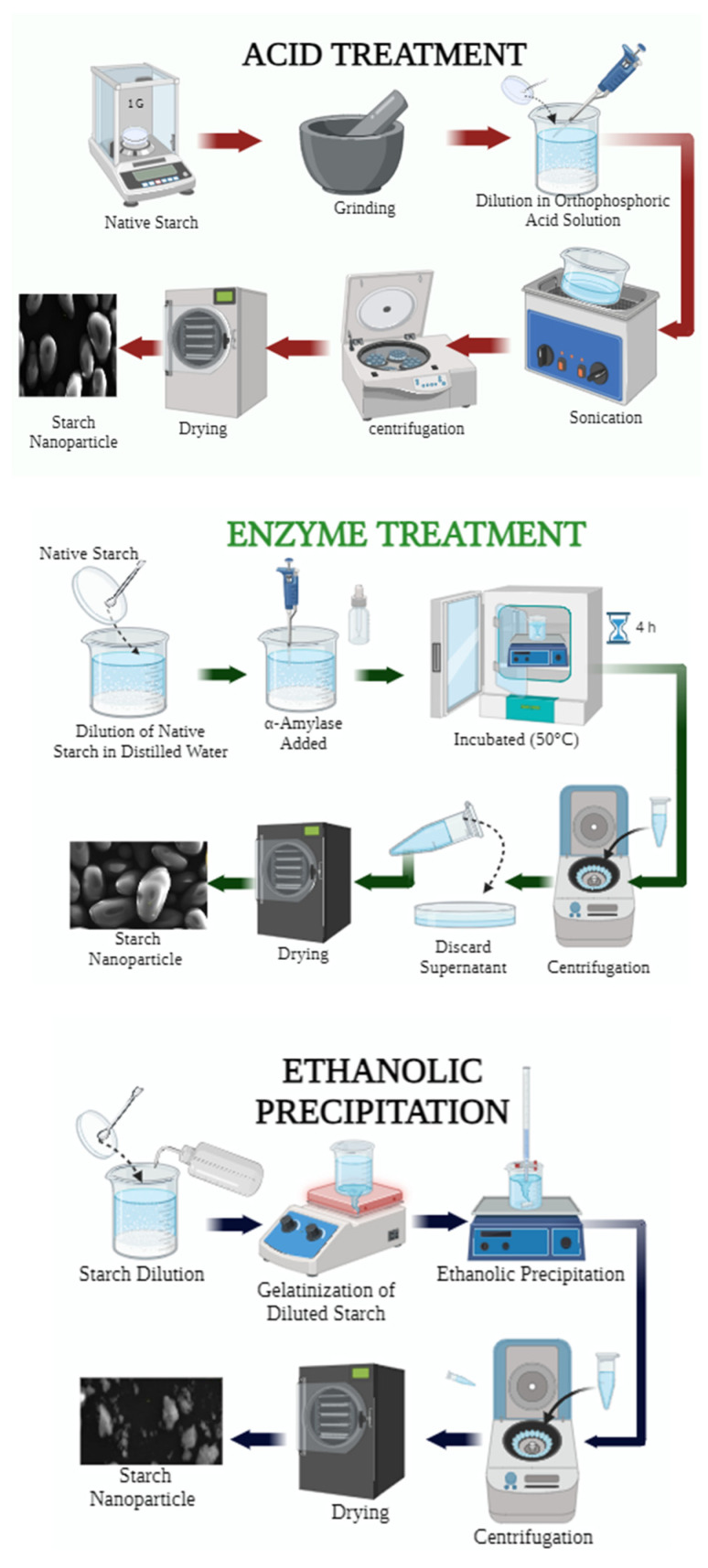
Starch modification by acid, enzymatic treatment, and ethanolic precipitation.

The method described by Qin et al. [27] was followed, involving the gelatinization of starch followed by precipitation with ethanol (96% purity, Alkofarma, Lima, Peru). Two grams of starch were dispersed in 200 mL of distilled water and continuously stirred (CAT, Modelo M6, Staufen, Alemania) at 900 rpm at 80 °C for 30 min. The gelatinized starch was then cooled to room temperature and precipitated using 800 mL of 96% ethanol. Ethanol was added dropwise upon contact with the gelatinized starch while stirring at 700 rpm, gradually increasing the volume of alcohol until precipitation was complete. The precipitation process was maintained under agitation at 150 rpm for 18 h. Subsequently, the samples were centrifuged at (Centurión, 1.C2024, Chichester, UK) 3500 rpm for 10 min (twice) to remove the supernatant. Finally, the samples were vacuum dried (10 mBar) (BINDER, model VD56, Tuttlingen, Germany) at 20 °C for 4 h (Figure 1).

### 2.5. Color Analysis

The color assessment of the modified starch samples was conducted using a Konica Minolta benchtop colorimeter, model CR-5 (Tokyo, Japan). Colorimetric measurements were employed to ascertain lightness (L*, where 0 represents black and 100 represents white), hue angle (h*, Equation (1)), and chroma (C*, Equation (2)) by the standards set by the International Commission on Illumination (CIE). The yellowness index (YI) and the whiteness index (WI) were determined using Equations (3) and (4), respectively [28].

(1) $$h*=arctan\frac{b*}{a*}$$

(2) $$C=\sqrt{{a*}^{2}+{b*}^{2}}$$

(3) $$YI=\frac{142.86.b*}{L}$$

(4) $$WI=100-\sqrt{{\left(100-L\right)}^{2}+{\left(a*\right)}^{2}+{(b*)}^{2}}$$

### 2.6. Amylose and Amylopectin

The determination of amylose content was carried out utilizing a modified protocol based on the methodology proposed by Galicia et al. [29]. A 20 mg sample of starch was placed in a 50 mL screw-capped tube, into which 0.2 mL of 95% ethanol and 1.8 mL of 1 M sodium hydroxide were added to create a solution. The mixture stood for one day at room temperature, after which the volume was adjusted to 20 mL with deionized water and vigorously shaken for 30 min. Subsequently, a one-milliliter sample of the resulting solution was combined with two mL of 1 M acetic acid. Then, 0.4 mL of Lugol’s solution was added, and the volume was adjusted to 20 mL with deionized water and allowed to stand for 20 min. A portion of the solution was carefully withdrawn and transferred to a cuvette for absorbance measurement at a wavelength of 620 nm using a UV-Vis spectrophotometer. A standard curve was prepared using a 1 mg/mL amylose solution in deionized water to facilitate accurate quantification of the amylose content in the samples. The absorbance data were fitted to a linear equation, and the percentage of amylose was determined according to Equation (5).

(5) $$\%AM=\frac{M\cdot d}{f\cdot 100}$$

where M is the amylose (mg) obtained from the fitted equation, d is the dilution factor, and f is the mass of starch. 

The difference between total starch and amylose represents the amylopectin content.

### 2.7. Particle Size 

Next, 0.10 g of ethanolic precipitation-modified starch was suspended in 100 mL of ultrapure water and constantly stirred at 200 rpm for 24 h. Subsequently, the samples underwent ultrasound for 10 min at room temperature using a Sonics model VCX 750 sonicator (Newtown, CT, USA). An aliquot was extracted in a polystyrene cell, and the particle size distribution was determined by dynamic light scattering (DLS) using a Malvern brand instrument, model Zetasizer ZSU3100 (Worcestershire, UK).

### 2.8. Zeta Potential (ζ)

Four milligrams of the modified starch sample were dispersed in 5 mL of ultrapure water, stirred at 1000 rpm for 5 min, and then subjected to ultrasound for 10 min. Subsequently, 2 mL was taken and placed in a polystyrene cell in an apparatus (Malvern, model Zetasizer ZSU3100, Worcestershire, UK). Measurements were conducted using a laser with a wavelength of 632.8 nm, a scattering angle of −14.14°, and an electric field strength of 10 V/cm, with direct reading.

### 2.9. Swelling Power (SP), Water Solubility Index (WSI), and Water Absorption Index (WAI)

The procedure was performed following the guidelines outlined by Gani et al. [30], with certain modifications. A 0.2 g sample of modified starch (M_0_) was dispersed in 10 mL of distilled water. The swelling power, water solubility index, and water absorption index were calculated at 60, 70, and 80 °C. After manual shaking for 30 min, the mixture was centrifuged at 900× *g* for 30 min at room temperature using a centrifuge (Tom’s/Hampton-USA Science and Technology Corporation, model 033R-2). The supernatant was then carefully removed from the Petri dishes, and the swollen starch granules (M_1_) were weighed. The supernatant was dried for four hours at a temperature of 90 °C until reaching constant weight (M_2_). SP, WSI, and WAI values were calculated using Equations (6), (7), and (8), respectively.

(6) $$SP\left(\frac{g_{swollen}}{g_{starch}-g_{soluble}}\right)=\frac{M_{1}}{M_{0}-M_{2}}$$

(7) $$WSI\left(\frac{g_{soluble}}{100g}\right)=\frac{M_{2}}{M_{0}}$$

(8) $$WAI\left(\frac{g_{swollen}}{100g}\right)=\frac{M_{1}}{M_{0}}$$

### 2.10. Paste Properties

Paste property analysis was carried out using an Anton Paar rotational rheometer (Model MCR 702e, Graz, Austria) equipped with a starch cell attachment. Initially, the water content of the starch was determined, and a 10% *w*/*w* suspension was prepared and allowed to stand for 60 min. Subsequently, 20 g of the suspension was weighed and introduced to the starch cell. The experimental procedure consisted of an initial agitation at 600 rpm for 20 s, followed by a constant speed of 160 rpm throughout the test. This protocol included a 2 min stabilization phase at 50 °C, followed by heating at 6 °C/min to 95 °C, a 5 min isothermal hold at 95 °C, cooling at 6 °C/min to 50 °C, and finally an isothermal retention of 2 min at 50 °C. Parameters such as paste temperature, maximum viscosity, minimum viscosity, final viscosity, burst viscosity, and recovery viscosity were recorded using Reocompass© software V1.30.999.Release. To ensure the accuracy and reliability of the results, all measurements were performed in duplicate.

### 2.11. X-ray Diffraction

X-ray diffraction measurements were performed on modified starch samples using an X-ray diffractometer, X’Pert Pro (PANalytical, Almelo, The Netherlands). CuKa-1 radiation at 40 mA and 40 kV was used. Data were obtained by scanning over a 2θ interval at room temperature, specifically between 5 and 50 °C. Two grams of sample were taken and adjusted to a moisture content of 4% prior to analysis. For analysis, the starch sample was placed in a PANalytical universal powder sample holder. Origin Pro v.2022 software was used to determine the crystalline and amorphous regions of the modified starches, successfully separating them. The relative crystallinity was then calculated by dividing the crystalline peak area by the total diffraction area obtained.

### 2.12. Thermal Analysis

The thermal stability of the modified starch samples was evaluated by two methods: thermogravimetric analysis (TGA) and differential scanning calorimetry (DSC). In TGA, the sample was loaded into an alumina crucible (Al_2_O_3_) and analyzed using a calorimetric analyzer (TA Instruments, TGA550, New Castle, DE, USA). Spectra were recorded using Trios V5.0.0.44616T software, with a temperature range of 20 to 600 °C, a heating rate of 10 °C/min, and a nitrogen flow rate of 50 mL/min. Differential scanning calorimetry (DSC) (DSC2500, TA Instruments, New Castle, DE, USA) was used to determine the thermal conversion parameters in a nitrogen atmosphere (50 mL/min). The modified starch samples were hermetically sealed in aluminum containers and heated from 20 to 200 °C at a heating rate of 5 °C/min. The initial DSC measurement was performed using an empty plate as a reference under the same conditions as for the sample measurement. The spectra were recorded using Trios V5.0.0.44616 software.

### 2.13. FT-IR Spectra

Tablets containing a 0.1% concentration of KBr-modified starch (IR grade, Darmstadt, Germany) were prepared and loaded into the transmission module of a Thermo Fisher Nicolet IS50 (Waltham, MA, USA) FT-IR (Fourier transform IR spectroscopy) spectrometer. The wavenumber range used was 4000 to 400 cm^−1^, with a resolution of 4 cm^−1^.

### 2.14. SEM Morphology

A Quanta model 200 scanning electron microscope (SEM, Thermo Fisher, Waltham, MA, USA) was used to analyze the modified starch samples. The starch granules were fixed directly onto a conductive carbon tape, ensuring stability during analysis. Each sample was introduced into the SEM vacuum chamber and measured at an accelerating voltage of 25 kV and 1000× magnification. The SEM software AutoScript 4 enabled image tracing and quantitative measurements of the morphology of the modified starch to be obtained.

### 2.15. Statistical Analysis

The results obtained were analyzed using the general linear model ANOVA with Tukey’s multiple comparisons test with a confidence level of 95% in the trial version of the Minitab software v.20. Tables were organized in Microsoft Excel, and figures were created with Origin Pro software.

## 3. Results and Discussion

### 3.1. Color Analysis

In the food industry, color is considered an important parameter, as consumers usually prefer high brightness values [31]. The standard color of potato starches is white or a light cream tone, which can vary depending on the process of obtaining and/or treating the starch to modify its characteristics. In this study, significant differences were found in the colorimetric values of the modified starch samples analyzed, (*p*-value < 0.05) (Table 1).

These modifications darkened the starch and reduced its reddish and yellowish hues. This is due to the double-helix structure of amylopectin, which when broken forms complexes with impurities such as proteins, lipids, and fibers present in the samples [32], which is confirmed in Table 1, as an increased angle of the first quadrant of the CIElab space (towards yellow), with greater emphasis for the acid treatment. In previous studies, the L* value of pinhao starch has been reported to be 95.48 ± 0.15 but to decrease to 94.98 ± 0.05 with acid treatment and to 94.69 ± 0.07 with ultrasound [33]; furthermore, Jhan et al. [31] found that the L* value of millet starch decreases from 91.33 ± 0.72 to 88.88 ± 0.19 when nano-reduced starch is obtained. 

**Figure 2:**
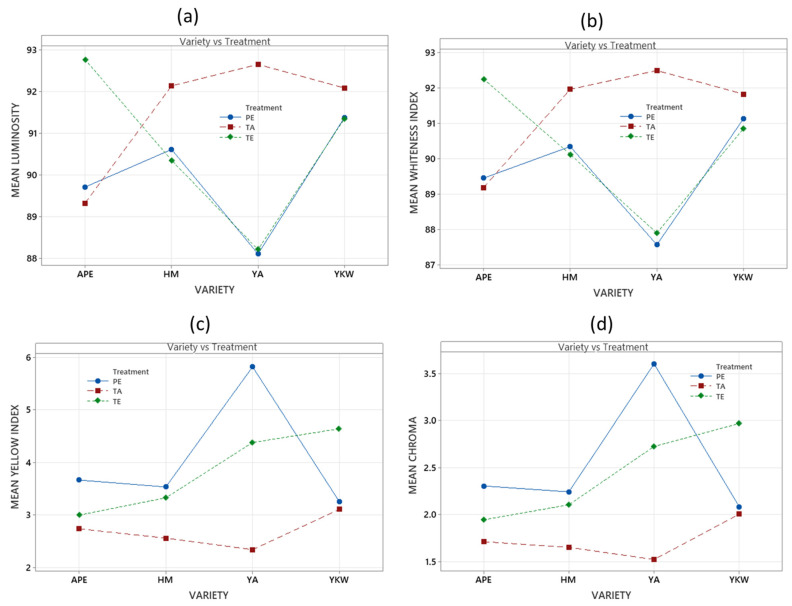
Color attributes by CIElab space, luminosity (**a**); whiteness index (**b**); yellowness index (**c**); and chroma (**d**).

In their study, Dey and Sit [32] found a significant reduction in the luminosity of modified foxtail millet starch compared to that of native starch, which coincides with the results of this work (Table 1). Regarding the treatments applied for starch modification, a decrease in L* and WI values was observed in the Yurac Anca and Huarmi Mallco varieties when ethanolic precipitation and enzymatic treatment were used. However, the opposite occurred when acid treatment was applied, since a higher value was obtained (Figure 2a,b) in these two varieties. Color intensity, C*, and yellow index, YI, should have low values in modified starch. The acid treatment yielded lower values of YI and C* (Figure 2c,d) for all four varieties.

### 3.2. Amylose/Amylopectin Content, z-Potential (ζ)

Highly efficient nanocarriers and drug coatings can obtain excellent properties by modifying starch by reducing its amylose content and generating positive surface charges [34].

An investigation by Bajer [35] focused on the production of nanostarch for food applications by acid hydrolysis methods with HCl and sonication, as well as from both methods combined, and amylose losses were observed in corn starch, indicating an increased susceptibility of unbranched macrochains (occurring mainly in amylose) to HCl and ultrasound treatment. However, this could not be evidenced in this study (Table 2), with the results presenting an opposite effect in potato varieties APE, YKW, YA, and HM; this phenomenon could be attributed to the fact that there was no rupture of the amylose chains by the action of the acid. In another study, Torres et al. [36] explored the tailoring of potato starch nanoparticle properties by nanoprecipitation (NP), alkaline hydrolysis, and nanoprecipitation with crosslinking agent (NPC), and the results indicated that the amylose content ranged from 13.71% to 21.39% for NP, 12.16% for alkaline hydrolysis, and 20.72% for NPC. This suggests that nanoprecipitation and alkaline hydrolysis can significantly alter amylose content depending on the specific modification method used. In the present study, similar behavior was found for treatment by ethanolic precipitation, yielding an amylose content ranging from 28 to 38%, lower than that found in the other treatments. Gujral et al. [37] studied the synthesis and characterization of potato starch nanoparticles exposed to sulfuric acid for 7 days, and the results showed that the amylose content became undetectable, indicating that extensive hydrolysis eliminates the presence of amylose in the modified starch.

**Figure 3:**
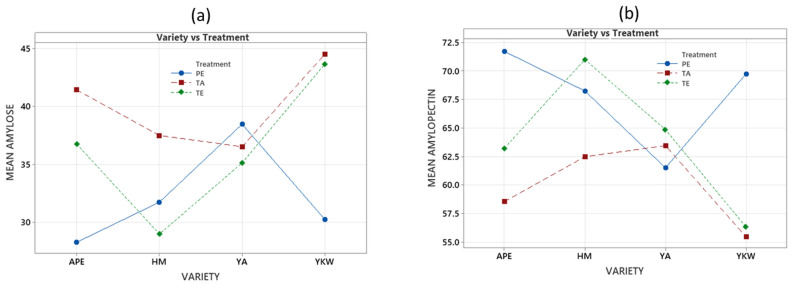
Graph of interactions for PE ethanolic precipitation, TA acid treatment, and TE enzymatic treatment with amylose (**a**) and amylopectin (**b**).

Meanwhile, Li et al. [38] investigated the structural and physicochemical properties of granular starches after treatment with enzymatic hydrolysis. The results showed that the amylose content remained relatively high, between 37.5 and 40.6%. This is a similar result to that found in the present study, which presented a variation between 29 and 43% for all varieties. This suggests that enzymatic hydrolysis is suitable for applications where a high amylose content is required. In conclusion, the amylose content of modified starches varies significantly depending on the treatment method and the potato variety of the starch (Figure 3).

A high zeta potential increases the electrostatic repulsions between particles, resulting in a decrease in the Van der Waals forces of attraction that are responsible for particle agglomeration, eventually leading to larger particles [23]. The zeta potential (ζ) of the modified starches in this study showed that the enzymatic treatment yielded higher values, ranging between −21.28 and −26.55 mV, followed by the acid treatment with values between −22.29 and −26.36 mV. On the other hand, ethanolic precipitation resulted in lower values, ranging between −10.13 and −17.94 mV (Table 2). These results are consistent with those reported by Wen et al. [39] for modified cassava starches, with zeta potential values between −17 and −25.38 mV, and are also lower than the values of −41.29 mV reported by [23].

### 3.3. Swelling Power (PH), Water Solubility Index (WSI), and Water Absorption Index (WAI)

The results concerning the swelling power (SP), water solubility index (WSI), and water absorption index (WAI) of native and modified starches evaluated at a temperature of 80 °C are detailed in Table 3. In general, the various treatments used for starch modification resulted in a decrease in WAI, an increase in WSI, and a variation in SP behavior. The treatments applied to modify starch have been shown to impact its properties. The degree of starch granule breakdown, amylose leaching, and amylopectin partitioning influences the ability of the sample to form a gel [34].

**Figure 4:**
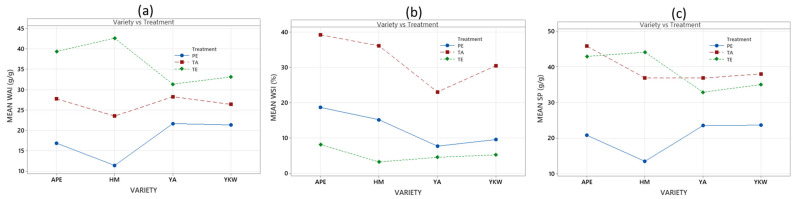
Interactions of factors for techno-functional properties, where PE is ethanolic precipitation, TA is acid treatment, and TE is enzymatic treatment, with the water absorption index (**a**), water solubility index (**b**) and swelling power (**c**).

A pronounced reduction in SP was observed with ethanolic precipitation treatment for all potato starch varieties. It is likely that due to the bonds between long amylopectin chains, the low swelling power may be related to the formation of crystals [40]. On the other hand, there is an interaction between the factors studied, with greater emphasis on the HM variety with the different treatments (Figure 4c). The acid treatment presented a behavior similar to that of native starch (Table 3).

The water solubility index (WSI) is a parameter that indicates the amount of compounds released from the gel and is often used as an indicator of biopolymer degradation and the degree of starch dextrinization [34]. The acid treatment presented higher WSI values, and significant differences were found (Tukey’s test with 95% confidence), with a strong interaction between the acid treatment and the different varieties studied (Figure 4b). The increase in WSI caused by acid treatment suggests higher solubility and good hydration of the molecules, which promotes high transparency [41]. In addition, strong depolymerization could increase the content of soluble compounds and thus increase WSI values [34]. On the other hand, the decrease in WSI upon enzymatic treatment could be attributed to the internal rearrangement or reassociation of starch granules [41]; in addition, they presented a weak interaction between the studied factors (Figure 4b). An increase in solubility and an increase in swelling power are associated with water absorption capacity, which is corroborated by the results obtained in the present study (Figure 4a,c).

The reduction in WAI may be related to a change in the organization of starch granules due to the dissipation of energy transmitted by ultrasound [34]; this is confirmed by the results obtained for the acid treatment using ultrasound (Figure 4a). It was also observed that the enzymatic treatment yielded a higher WAI in all the varieties studied, especially in the HM variety, possibly due to a high association strength in the starch granules that led to a lower WSI value (Figure 4b).

On the other hand, the strength of association refers to the interaction between amylose and amylopectin molecules within the starch granules, since a lower capacity to retain water and a higher solubilization may be indicative of a lower organization and strength of association in the starch granules. The modification of starch with ethanolic precipitation caused a lower WAI for all the varieties studied and also presented a strong interaction between the factors studied (Figure 4a).

### 3.4. Paste Properties

**Figure 5:**
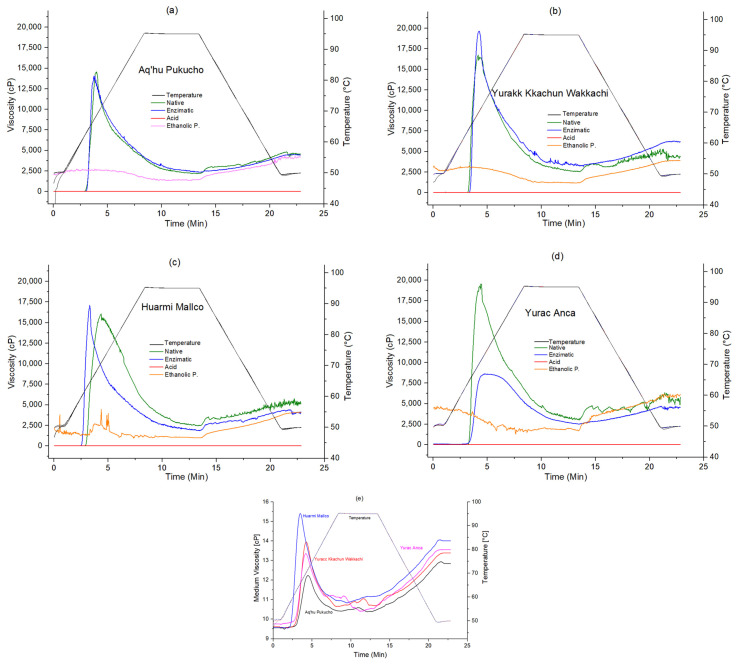
Paste properties and apparent viscosity curves of native and modified starches, being (**a**) APE, (**b**) YKW, (**c**) HM, (**d**) YA and, (**e**) apparent viscosity curves of acid treatment.

Figure 5 shows that native and modified potato starches from four native varieties were subjected to heating and cooling cycles, resulting in gelatinization, gelation, retrogradation, or none of these to a high or lesser degree.

The sticking curve of starch modified by ethanolic precipitation showed an unusual shape without the characteristic receding and breaking regions (Figure 5a–d), possibly due to changes in its structure. The final viscosity curve was observed to be close to the viscosity curves of native starches, indicating that ethanolic precipitation gelatinized the granules. Normally, starch granules are insoluble in cold water and only form suspensions; however, hydrolysis due to ethanolic precipitation treatment breaks the starch granule structure, producing low-molecular-weight dextrin [42] of nanometer size. Therefore, the modified starch solution obtained by ethanolic precipitation showed a reduction in the paste property parameters Pv, Bd, Sb, and Fv (Figure 5), resulting in higher stability, clarity, binding properties.

The acid treatment significantly decreased viscosity (Figure 5a–d); however, typical paste property curves were seen for these starches (Figure 5e), in which the parameters Pv, Bd, Sb, and Fv can be identified. This may be because acid treatment tends to hydrolyze the starch structure into more linear segments, decreasing viscosity with heat treatment and increasing its cold solubility [43]. Likewise, Pizarro et al. [44] found that the higher amylose content decreases the viscosity of starches, presenting a similar result to that of the acid treatment (Table 2). These starches exhibit typical viscous behavior, but due to their very low viscosity, only starches modified enzymatically and by ethanolic precipitation were analyzed. Aq’hu Pukucho, Yurakk Kkachun Wakkachi, and Huarmi Mallco starches appear to be more resistant to the action of the enzyme used, whereas Yurac Anca starch was partially affected but showed a final viscosity similar to that of native starch (Figure 5d).

Paste temperature did not show significant differences (*p* < 0.05); all treatments showed the same thermal resistance during swelling. The viscosity peak reflects the equilibrium between the swelling and breaking of the starch granules. The highest values corresponded to starches modified by enzymatic treatment, followed by ethanolic precipitation (Table 4). The stability of hot and cold paste is evidenced by the lower decomposition viscosity Bd and starch Sb regression [45]. The Bd, referring to the disintegration of swollen starch granules during cooling, was lower for ethanolic precipitation than for enzymatic treatment, suggesting higher hot and cold paste stability [45] with better granule shear strength [46] for starches modified by ethanolic precipitation. Final viscosity, Fv, indicates the ability to form viscous pastes due to amylose retrogradation upon cooling; low values indicate higher resistance of the gel formed, and ethanolic precipitation obtained lower values than the enzymatic treatment (Table 4).

The applied modifications altered the structure and properties of the starches, decreasing the viscosity measured by RVA. On the other hand, they improved the paste capacity, which made the ethanolic precipitation treatment gelatinize more easily at lower temperatures, resulting in lower viscosity compared to native starches (Figure 5).

### 3.5. X-ray Diffraction

X-ray diffraction diagrams are used to analyze the crystalline structure of materials. Starches present semicrystalline structures and amorphous structures, and their relationship defines the behavior of starch in different applications; these structures result in a typical X-ray diffraction pattern.

**Figure 6:**
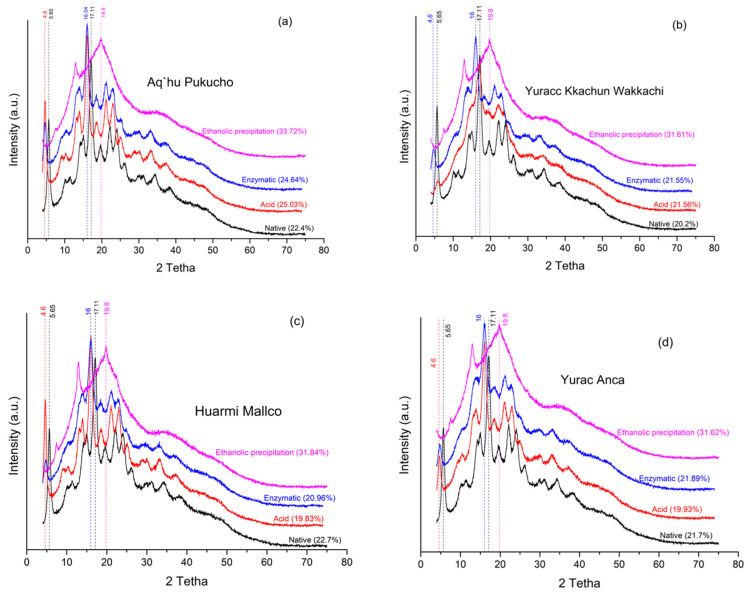
X-ray diffraction for native starch, modified starch, and percent crystallinity, being (**a**) APE, (**b**) YKW, (**c**) HM, and (**d**) YA.

The native starch of the Aq’hu Pukucho (APE) variety shows several peaks in the X-ray diffraction diagram. These peaks were located at angles of 2θ = 5.65° (type B), 15.01°, 17.11°, 19.51°, and 24.08° (type C). However, upon the application of acid and enzymatic treatments, these peaks changed their position. For example, the peak at 2θ = 5.65° shifted to 4.60° after acid and enzymatic treatments, while it disappeared completely with ethanolic precipitation. Similarly, the peak at 17.11° shifted to 16.04° in the acid and enzymatic treatments, and ethanolic precipitation produced a broader and less intense peak at 19.8°. This behavior was similar for all starch varieties (Figure 6b–d). These results indicate that the treatments applied to modify native starch also altered its crystalline structure.

Enzymatic hydrolysis affects not only the amorphous region of starch but also the crystalline region, as mentioned in a study by [1]. In our study, we observed that acid and enzymatic treatments modified the X-ray diffraction pattern in the bitter varieties Huarmi Mallco and Yurac Anca, resulting in a decrease in crystalline components (Figure 6a,c). On the other hand, ethanolic precipitation decreased the amorphous region, causing an increase in the crystalline region in all the varieties investigated (Figure 6a–d).

### 3.6. Thermogravimetric Analysis (TGA)

**Figure 7:**
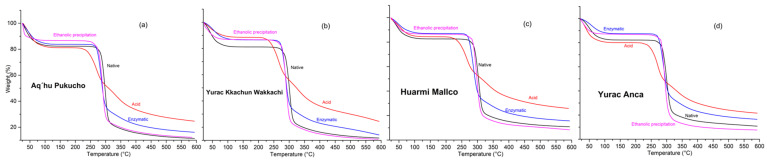
TGA of native and modified potato starch varieties APE (**a**), YKW (**b**), HM (**c**), and YA (**d**).

The thermogravimetric curves for both native and modified starch (Figure 7) show three main stages of mass loss and four stages in the case of acid treatment. The first stage is desiccation, which begins as temperature increases and ends between 234 and 240 °C for ethanolic precipitation, enzymatic treatment, and native starch; in the case of acid treatment, this stage occurs between 161 and 199 °C. The percentage of mass loss in this stage is attributed to moisture release and the thermal degradation of the polymer [47] due to the water content of the samples. The second stage is the main degradation, which ends at approximately 339 °C (Table 5). During this stage of starch pyrolysis, substances such as water, carbon dioxide, carbon monoxide, acetaldehyde, furan, and 2-methylfuran are released [48]. This thermal decomposition is generally considered an important process associated with amylose and amylopectin degradation mechanisms. The third stage ends with the formation of black smoke, which occurs between 587 and 592 °C for native starch, enzymatic treatment, and ethanol precipitation and between 369 and 592 °C for acid treatment.

Starch modified with ethanolic precipitation (E.P.) had a lower residual mass (Table 5) (Figure 7a–d), generally related to the inorganic residue remaining after polymer decomposition [47].

### 3.7. Thermal Stability Analysis by DSC

**Figure 8:**
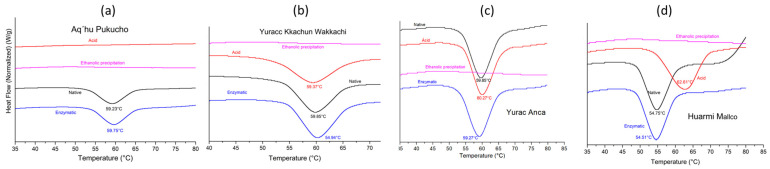
Gelatinization temperatures of native and modified starch varieties APE (**a**), YKW (**b**), HM (**c**), YA (**d**).

DSC thermograms of native and modified starches showed endothermic gelatinization peaks, which represent the energy required to break the double-helix structure during this process [49]. It was observed that starches modified by ethanolic precipitation and acid treatment in the APE variety presented minimum enthalpy values (Figure 8). This lower enthalpy compared to that of native starch indicates a reduction in the double-helix content in both the crystalline and non-crystalline areas [49]. It has been suggested that this could be due to the gelatinization of starch during treatment, resulting in a lower gelatinization temperature [39] (Table 6).

### 3.8. Fourier Transform IR Spectroscopy

**Figure 9:**
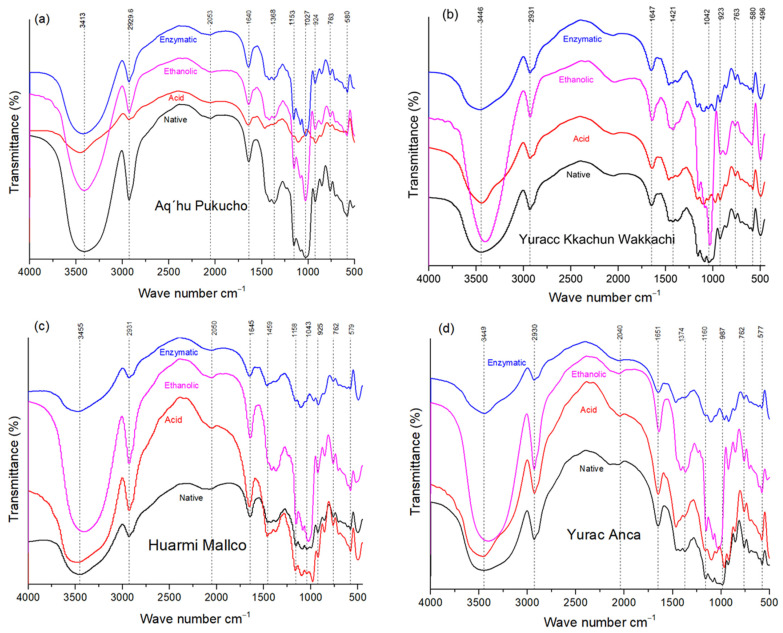
FT-IR spectra of native and modified native potato starches for APE, Aq’hu Pukucho (**a**); YKW, Yurakk Kkachun Wakkachi (**b**); HM, Huarmi Mallco (**c**); and YA, Yurac Anca (**d**).

FT-IR spectroscopy analyzes representative chemical bonds as infrared radiation passes through a sample [50]. The FT-IR spectra of native and modified native potato starches are illustrated in Figure 9, where it is observed that the shapes of the spectra of the native starch samples and the modified starches are similar, but considerable variations in the intensity of the bands is observed.

This phenomenon is probably attributed to the combined effect of reducing the particle size and increasing the number of molecules present. Smaller particles generally have lower porosity, which leads to denser packing of molecules within the sample. With a higher concentration of molecules in the infrared radiation path, there is greater absorption, resulting in more intense spectral bands.

It could be noticed that in stretching bands between 3413–3455 cm^−1^, corresponding to the stretching vibrations of O-H bonds, present in the hydroxyl groups of glucose monomers forming starch [51], the modified starch samples presented higher intensity in this range (Figure 9). Generally, in gelatinized starches, this band is dislocated for higher wavenumbers by the elimination of two hydroxyl groups [52]. Although these samples were heat treated, this process was not sufficient for the removal of hydroxyl groups. It is noticeable that the vibration in the range of 1027 to 1040 cm^−1^ was more intense for ethanolic precipitation (Figure 9).

The sharp band at 2932 cm ^−1^ is characteristic of the C-H stretches associated with the hydrogen atoms of the ring, and higher band intensity was observed in starches modified by ethanolic precipitation and acid treatment than in native starch from Huarmi Mallco and Yurac Anca varieties. The intensity changes in the C-H stretching range could be attributed to the change in the amylose and amylopectin content of the starch molecule [53]

### 3.9. Morphology SEM

**Figure 10:**
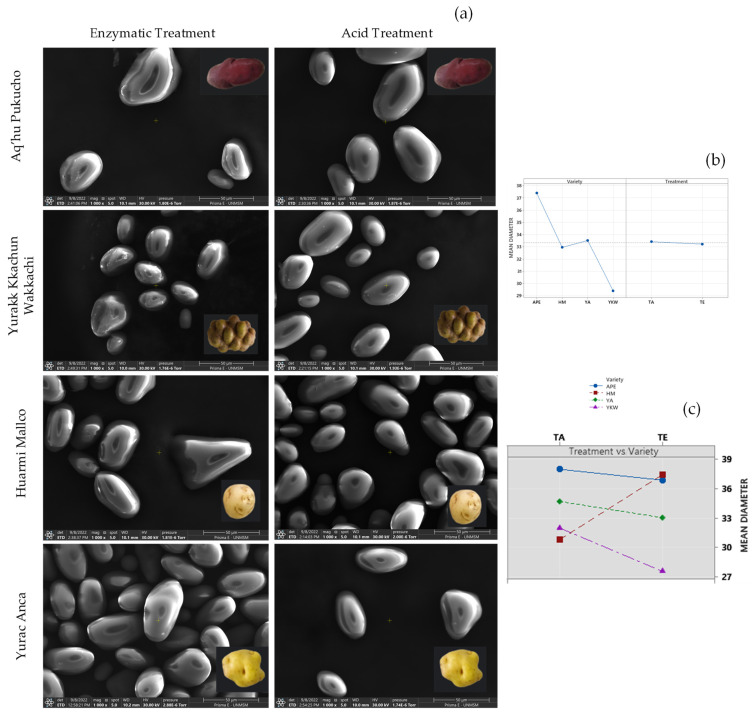
Photomicrograph of starches (×1000 and 50 μm) with enzymatic and acid treatment (**a**), main effects for particle size (**b**), and interaction effects for particle size (**c**).

SEM microphotographs showed that the starch granules preserved their structural integrity in the face of acid and enzymatic treatments, retaining their elongated spherical and ellipsoid shape (Figure 10a), with a diameter ranging from 27.52 to 37.96 µm (Table 7). Unlike what was observed by Sujka and Jamroz [54] regarding the effects of ultrasonic action on potato starch granules, where cracks and depressions on the surface were identified, this phenomenon was not evident in the present study in the samples subjected to acid treatment assisted by ultrasound.

Regarding particle size, it was observed that the effect of the variety was more significant than the effect of the modification treatment (Figure 10b). Likewise, an interaction of factors was found for the Huarmi Mallco variety, where the particle size was smaller with the acid treatment (Figure 10c).

**Figure 11:**
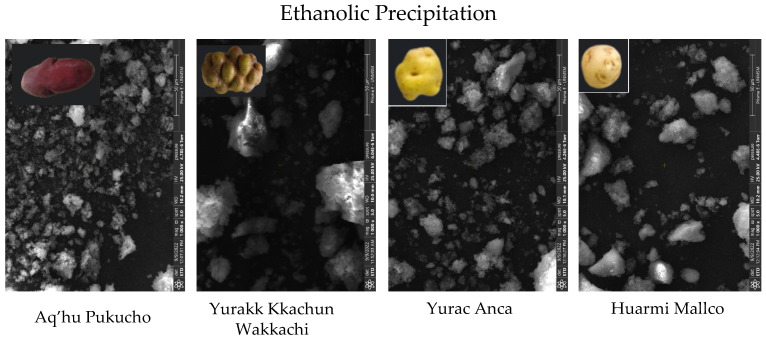
Microphotography of starches (×1000 and 50 μm) with ethanolic precipitation.

Disparities were detected between SEM images taken from ethanolic precipitation-treated samples, which showed a cloudy structure. These findings confirm the results of Kaul et al. [55], who point out that pregelatinized starches undergo complete and irregular structural changes. Figure 11 and Table 8 illustrate the size distributions of starches treated with ethanolic precipitation, as determined by DLS.

The mean size of starches treated by the ethanolic precipitation method presented three peaks in the four varieties studied, suggesting that the particle size distribution is not homogeneous, being 23.1 nm (81.83%) for the HM variety, 10.9 nm (92%) for the YA variety, 606.8 nm (77.1%) for the YKW variety, and 476.3 nm (75.76%) for the APE variety, with the Yurac Anca (YA) variety presenting the smallest size (Table 8). Recent research reports obtaining nanostarch of sizes ranging from 30 to 600 nm [56,57], depending on the nature of the native starch. Moreover, the type of starch influences the final size of the nanostarch.

## 4. Conclusions

Modified starch obtained from native potatoes is seen as a promising raw material for the manufacture of biocomposites for the food and pharmaceutical industries. Among the modification techniques employed, ethanol precipitation has stood out for its ability to significantly reduce the amylose content compared to other methods. On the other hand, enzymatic hydrolysis has been shown to maintain relatively high levels of amylose, suggesting its relevance for applications that demand a high content of this component. As for the changes generated by starch modification, ethanol precipitation induced a decrease in the water absorption index (WAI) in all the varieties studied, showing a marked interaction between the factors involved. Likewise, this technique has caused alterations in both the structure and properties of starch, with a notable reduction in viscosity measured by RVA. Despite this decrease in viscosity, an improvement in the ability of the modified starch to form a paste was observed, suggesting greater stability during gelatinization, both at high and low temperatures, as well as greater strength of the resulting gels. On the other hand, acid treatment generated a decrease in starch viscosity, although it maintained the typical characteristics of starch paste. In addition, both acid and enzymatic treatments produced modifications in the X-ray diffraction patterns in certain varieties, with a consequent decrease in the crystalline components. In contrast, ethanol precipitation reduced the amorphous region and led to an increase in the crystalline region in all the varieties studied. Acid treatment was found to confer higher thermal stability compared to enzymatic hydrolysis and ethanol precipitation, and exceptionally low gelatinization temperatures were detected by DSC. Also, ethanol precipitation altered the morphology of native starch, as demonstrated by scanning electron microscopy (SEM) images and nanometer particle sizes (DLS).

These characteristics suggest a broad application potential for modified starches, without the need for additional heat treatment.

## Figures and Tables

**Table 1:** Color parameters of modified starch obtained by different treatments.

Treatment	Variety	Color Attributes of Modified Starch				
		Luminosity (L*)	Hue (h*)	Chroma (C*)	Whiteness Index (WI) %	Yellow Index (YI)
		$\bar{x}$ ± SD *	$\bar{x}$ ± SD *	$\bar{x}$ ± SD *	$\bar{x}$ ± SD *	$\bar{x}$ ± SD *
Acid	APE	89.33 ± 0.03 ^ab^	92.01 ± 0.05 ^bc^	1.71 ± 0.05 ^ef^	89.19 ± 0.03 ^abc^	2.74 ± 0.07 ^efg^
	YKW	92.08 ± 0.46 ^ab^	93.39 ± 2.44 ^ab^	2.01 ± 0.54 ^de^	91.82 ± 0.57 ^ab^	3.11 ± 0.85 ^def^
	HM	92.13 ± 0.03 ^ab^	91.62 ± 0.21 ^bc^	1.65 ± 0.01 ^ef^	91.96 ± 0.02 ^ab^	2.56 ± 0.01 ^fg^
	YA	92.64 ± 0.02 ^ab^	94.51 ± 0.00 ^a^	1.52 ± 0.00 ^f^	92.49 ± 0.02 ^a^	2.34 ± 0.00 ^g^
Enzymatic	APE	92.76 ± 5.31 ^a^	90.02 ± 0.00 ^cde^	1.95 ± 0.08 ^def^	92.25 ± 4.73 ª	3.00 ± 0.15 ^defg^
	YKW	91.35 ± 0.02 ^ab^	90.02 ± 0.83 ^def^	2.97 ± 0.02 ^b^	90.85 ± 0.01 ^abc^	4.64 ± 0.03 ^b^
	HM	90.61 ± 0.03 ^ab^	90.02 ± 0.00 ^cd^	2.24 ± 0.01 ^de^	90.35 ± 0.03 ^abc^	3.54 ± 0.01 ^de^
	YA	88.22 ± 0.05 ^b^	90.02 ± 0.11 ^h^	2.73 ± 0.01 ^bc^	87.91 ± 0.04 ^bc^	4.38 ± 0.02 ^bc^
Ethanolic Precipitation	APE	89.71 ± 0.06 ^ab^	90.02 ± 0.48 ^fg^	2.31 ± 0.03 ^cd^	89.45 ± 0.05 ^abc^	3.67 ± 0.04 ^cd^
	YKW	91.38 ± 0.08 ^ab^	90.02 ± 0.14 ^ef^	2.08 ± 0.07 ^de^	91.13 ± 0.08 ^abc^	3.26 ± 0.10 ^def^
	HM	90.61 ± 0.03 ^ab^	90.02 ± 0.00 ^def^	2.24 ± 0.01 ^d^	90.35 ± 0.03 ^abc^	3.54 ± 0.01 ^d^
	YA	88.11 ± 0.17 ^b^	90.02 ± 0.14 ^gh^	3.60 ± 0.06 ^a^	87.58 ± 0.18 ^c^	5.82 ± 0.11 ^a^
Native	APE	92.68 ± 0.00	85.40 ± 0.30	4.32 ± 0.04	91.50 ± 0.02	6.64 ± 0.07
	YKW	92.71 ± 0.01	87.68 ± 0.00	4.95 ± 0.01	91.19 ± 0.01	7.62 ± 0.01
	HM	91.23 ± 0.07	91.16 ± 0.34	4.09 ± 0.08	90.32 ± 0.08	6.41 ± 0.12
	YA	90.73 ± 0.09	86.35 ± 0.28	3.61 ± 0.03	90.05 ± 0.08	5.67 ± 0.04

Where $\bar{x}$ is arithmetic mean; SD is standard deviation; * shows significance; and different letters in a row indicate a significant difference, evaluated with Tukey’s test at 95% confidence.

**Table 2:** Amylose/amylopectin composition and ζ of the modified starch.

Component	Native Potato Variety	Treatment		
		Acid	Enzymatic	Ethanolic Precipitation
		$\bar{x}$ ± SD *	$\bar{x}$ ± SD *	$\bar{x}$ ± SD *
Amylose	APE	41.43 ± 0.25 ^c^	36.76 ± 0.18 ^f^	28.29 ± 0.25 ^k^
	YKW	44.51 ± 0.15 ^a^	43.66 ± 0.25 ^b^	30.25 ± 0.19 ^i^
	YA	36.53 ± 0.29 ^f^	35.13 ± 0.22 ^g^	38.47 ± 0.34 ^d^
	HM	37.50 ± 0.14 ^e^	29.00 ± 0.18 ^j^	31.74 ± 0.19 ^h^
Amylopectin	APE	58.57 ± 0.25 ^i^	63.24 ± 0.18 ^f^	71.71 ± 0.25 ^a^
	YKW	55.49 ± 0.15 ^k^	56.34 ± 0.25 ^j^	69.75 ± 0.19 ^c^
	YA	63.47 ± 0.29 ^f^	64.87 ± 0.22 ^e^	61.53 ± 0.34 ^h^
	HM	62.50 ± 0.14 ^g^	71.00 ± 0.18 ^b^	68.26 ± 0.19 ^d^
z-Potential	APE	−25.04	−31.55	−17.69
	YKW	−26.36	−26.55	−17.94
	YA	−22.29	−25.5	−17.08
	HM	−25.82	−21.28	−10.13

Where $\bar{x}$ is arithmetic mean; SD is standard deviation; * is significance; and different letters in a row indicate a significant difference, evaluated with Tukey’s test at 95% confidence.

**Table 3:** Techno-functional properties of native and modified starches.

Treatment	Variety	WAI (g/g)	WSI (%)	SP (g/g)
		$\bar{x}$ ± SD *	$\bar{x}$ ± SD *	$\bar{x}$ ± SD *
Ethanolic Precipitation	APE	16.90 ± 1.42 ^fg^	18.71 ± 3.15 ^cd^	20.83 ± 2.25 ^e^
	YKW	21.38 ± 2.91 ^ef^	9.61 ± 0.37 ^ef^	23.66 ± 3.31 ^e^
	HM	11.46 ± 0.74 ^g^	20.23 ± 4.92 ^de^	14.83 ± 1.81 ^f^
	YA	21.70 ± 2.90 ^ef^	7.77 ± 0.48 ^fg^	23.53 ± 3.20 ^e^
Enzymatic	APE	39.39 ± 1.52 ^a^	6.43 ± 2.83 ^fg^	42.12 ± 1.81 ^abc^
	YKW	33.14 ± 0.67 ^b^	5.31 ± 0.39 ^fg^	35.00 ± 0.59 ^d^
	HM	42.64 ± 3.63 ^a^	3.30 ± 0.52 ^g^	44.09 ± 3.64 ^ab^
	YA	31.37 ± 3.16 ^bc^	4.60 ± 0.24 ^fg^	32.88 ± 3.39 ^d^
Acid	APE	27.77 ± 0.28 ^bcd^	39.21 ± 2.61 ^a^	45.75 ± 2.41 ^a^
	YKW	26.42 ± 0.42 ^cde^	30.48 ± 1.16 ^b^	38.01 ± 1.14 ^bcd^
	HM	23.55 ± 0.83 ^de^	36.12 ± 1.50 ^ab^	36.87 ± 0.85 ^cd^
	YA	28.29 ± 1.45 ^bcd^	21.24 ± 5.10 ^c^	36.09 ± 4.03 ^cd^
Native	APE	44.68 ± 1.25	2.55 ± 0.33	45.85 ± 1.14
	YKW	34.40 ± 0.69	7.59 ± 0.26	37.23 ± 0.69
	HM	34.41 ± 2.02	6.46 ± 0.72	36.78 ± 1.95
	YA	31.47 ± 0.33	5.26 ± 0.33	33.21 ± 0.42

Where $\bar{x}$ is arithmetic mean; SD is standard deviation; * shows significance; and different letters in a column indicate a significant difference, evaluated with Tukey’s test at 95% confidence.

**Table 4:** Pasting properties of modified potato starch.

Treatment	Variety	Pt (°C) ± SD *	Pv (cP) ± SD *	Bd (cP) ± SD *	Sb (cP) ± SD *	Fv (cP) ± SD *
Enzymatic	APE	63.765 ± 0.42 ^ab^	15,235.00 ± 1633.42 ^a^	13,035.00 ± 1859.69 ^a^	10,883.50 ± 1748.68 ^a^	4354.50 ± 112.43 ^ab^
	YKW	66.1 ± 1.54 ^ab^	17,095.00 ± 3599.17 ^a^	14,165.00 ± 3273.90 ^a^	11,491.00 ± 2784.59 ^a^	5602.00 ± 811.76 ^ab^
	HM	60.24 ± 0.69 ^abc^	16,665.00 ± 572.76 ^a^	14,812.50 ± 573.46 ^a^	12,628.50 ± 572.05 ^a^	4036.50 ± 0.71 ^b^
	YA	64.67 ± 1.07 ^ab^	8278.00 ± 507.70 ^b^	5795.50 ± 450.43 ^b^	3760.50 ± 471.64 ^b^	4517.50 ± 36.06 ^ab^
Acid	APE	60.91 ± 1.90 ^abc^	13.80 ± 1.49 ^c^	4.31 ± 1.71 ^c^	0.95 ± 0.25 ^bc^	12.85 ± 1.24 ^c^
	YKW	58.85 ± 1.11 ^abc^	14.79 ± 0.32 ^c^	4.82 ± 0.71 ^c^	1.80 ± 0.59 ^bc^	12.99 ± 0.28 ^c^
	HM	58.40 ± 0.79 ^bc^	15.89 ± 0.02 ^c^	6.02 ± 0.25 ^c^	1.93 ± 0.98 ^bc^	13.96 ± 1.00 ^c^
	YA	62.11 ± 2.14 ^abc^	17.63 ± 4.89 ^c^	8.30 ± 4.89 ^c^	4.65 ± 4.34 ^bc^	12.98 ± 0.56 ^c^
Ethanol Precipitation	APE	55.12 ± 1.29 ^c^	2399.00 ± 490.73 ^c^	1113.50 ± 419.31 ^c^	−2635.50 ± 1638.37 ^c^	5034.50 ± 1147.63 ^ab^
	YKW	58.77 ± 3.25 ^abc^	3624.50 ± 504.17 ^bc^	2352.00 ± 343.65 ^bc^	−784.50 ± 238.29 ^c^	4409.00 ± 742.46 ^ab^
	HM	59.33 ± 5.32 ^abc^	4221.00 ± 313.96 ^bc^	3266.50 ± 311.83 ^bc^	114.00 ± 339.41 ^bc^	4107.00 ± 25.46 ^b^
	YA	67.17 ± 0.88 ^a^	4385.50 ± 439.11 ^bc^	2996.50 ± 573.46 ^bc^	−1730.50 ± 426.39 ^c^	6116.00 ± 12.73 ^a^
Native	APE	60.30 ± 0.1	14,583 ± 113.7	12,413 ± 120.9	9928 ± 161.2	4656 ± 98.8
	YKW	62.67 ± 0.1	17,930 ± 1210.5	15,433 ± 1221.4	13,356 ± 980.3	4573 ± 224.6
	HM	60.90 ± 0.3	17,853 ± 1584.4	15,137 ± 1346.5	12,770 ± 1593.8	5086 ± 320.9
	YA	62.8 0 ± 0.2	19,450 ± 1772.1	16,447 ± 1721.9	13,536 ± 1483.8	5912 ± 320.9

Pasting temperature, Pt; peak viscosity, Pv; breakdown, Bd; setback viscosity, Sb; final viscosity, Fv; standard deviation, SD; significance, *; different letters in a column indicate a significant difference (*p* < 0.05).

**Table 5:** Weight loss and decomposition temperature of native and modified starches.

Treatment	Variety	Weight Loss								Residue
		First Stage		Second Stage		Third Stage		Fourth Stage		
		(%) ± SD	T (°C) ± SD	(%) ± SD	T (°C) ± SD	(%) ± SD	T (°C) ± SD	(%) ± SD	T (°C) ± SD	(%) ± SD
Native	APE	17.56 ± 0.32	239.84 ± 0.19	63.08 ± 0.89	339.96 ± 0.02	8.23 ± 0.25	592.43 ± 0.26	---------	---------	11.14 ± 0.32
	YKW	19.06 ± 0.11	240.01 ± 0.04	61.30 ± 1.06	339.99 ± 0.01	9.46 ± 1.10	590.52 ± 2.84	---------	---------	10.18 ± 2.23
	YA	17.86 ± 0.02	232.49 ± 1.03	62.84 ± 0.15	339.59 ± 0.12	10.8 ± 3.50	590.91 ± 1.73	---------	---------	8.50 ± 3.33
	HM	17.05 ± 0.62	239.31 ± 0.99	64.55 ± 2.11	339.93 ± 0.06	9.22 ± 2.30	591.85 ± 0.68	---------	---------	9.19 ± 0.81
Ethanolic Precipitation	APE	13.68 ± 0.20	224.91 ± 21.32	65.52 ± 0.87	339.00 ± 1.39	8.97 ± 0.41	583.57 ± 12.57	---------	---------	11.78 ± 0.62
	YKW	13.80 ± 0.36	239.67 ± 0.29	66.19 ± 1.45	339.97 ± 0.02	8.75 ± 0.47	592.56 ± 0.14	---------	---------	11.25 ± 0.61
	YA	13.03 ± 0.49	238.03 ± 0.09	72.11 ± 0.71	339.99 ± 0.02	6.76 ± 0.63	589.02 ± 0.43	---------	---------	8.06 ± 0.59
	HM	13.44 ± 0.47	234.55 ± 7.56	69.96 ± 0.03	339.53 ± 0.64	7.78 ± 0.22	587.20 ± 7.69	---------	---------	8.81 ± 0.73
Enzymatic	APE	16.19 ± 0.21	239.91 ± 0.00	56.42 ± 1.67	340.00 ± 0.00	12.01 ± 0.44	592.68 ± 0.03	---------	---------	15.36 ± 1.03
	YKW	13.28 ± 0.38	239.67 ± 0.29	58.94 ± 1.96	339.99 ± 0.01	13.28 ± 2.17	592.56 ± 0.14	---------	---------	14.49 ± 0.59
	YA	12.81 ± 0.26	240.03 ± 0.05	59.17 ± 2.86	339.99 ± 0.01	12.85 ± 1.21	596.15 ± 4.87	---------	---------	15.15 ± 1.92
	HM	13.10 ± 0.16	239.82 ± 0.12	57.68 ± 0.91	339.99 ± 0.01	13.22 ± 0.27	592.39 ± 0.35	---------	---------	15.99 ± 0.49
Acid	APE	18.55 ± 0.57	199.93 ± 0.16	31.02 ± 0.23	304.99 ± 0.00	13.90 ± 0.12	369.97 ± 0.01	12.09 ± 0.40	592.68 ± 0.03	24.43 ± 0.16
	YKW	11.03 ± 0.76	198.05 ± 2.85	33.90 ± 0.03	304.92 ± 0.12	14.89 ± 0.09	369.86 ± 0.21	14.47 ± 1.15	592.60 ± 0.42	25.67 ± 1.82
	YA	20.35 ± 0.45	161.62 ± 53.13	33.79 ± 0.46	304.62 ± 0.51	12.66 ± 0.04	369.41 ± 0.79	11.52 ± 0.05	590.68 ± 2.91	21.59 ± 0.01
	HM	15.45 ± 0.33	199.13 ± 1.33	33.18 ± 1.80	304.96 ± 0.05	13.91 ± 0.25	369.92 ± 0.06	12.60 ± 0.28	592.19 ± 0.46	24.84 ± 1.43

Where SD is standard deviation.

**Table 6:** Gelatinization temperatures of modified and native starches.

Treatment	Variety	°T Initial	°T Peak	°T Final	Residue
		$\bar{x}$ ± SD	$\bar{x}$ ± SD	$\bar{x}$ ± SD	
Native	APE	53.32 ± 0.25	59.25 ± 0.04	64.58 ± 0.20	3.63
	YKW	54.42 ± 0.05	59.85 ± 0.05	65.07 ± 0.05	3.62
	HM	48.94 ± 0.15	54.75 ± 0.09	59.92 ± 0.04	3.60
	YA	53.89 ± 0.11	59.85 ± 0.20	65.14 ± 0.08	4.30
Ethanolic Precipitation	APE	32.46 ± 0.06	34.50 ± 0.03	36.79 ± 0.19	--------
	YKW	33.15 ± 0.05	35.04 ± 0.05	37.05 ± 0.07	--------
	HM	33.39 ± 0.08	34.78 ± 0.07	37.48 ± 0.05	--------
	YA	32.47 ± 0.06	34.55 ± 0.07	40.30 ± 0.02	--------
Enzymatic	APE	53.66 ± 0.09	59.34 ± 0.04	64.44 ± 0.09	4.25
	YKW	54.69 ± 0.11	59.94 ± 0.05	65.11 ± 0.06	4.57
	HM	49.19 ± 0.14	54.51 ± 0.03	59.71 ± 0.08	3.77
	YA	52.74 ± 0.10	59.27 ± 0.06	64.79 ± 0.05	4.03
Acid	APE	33.77 ± 0.03	34.20 ± 1.75	36.71 ± 0.04	--------
	YKW	54.32 ± 0.07	59.37 ± 0.03	64.12 ± 0.07	3.20
	HM	54.14 ± 0.08	62.61 ± 0.06	69.15 ± 0.06	3.98
	YA	54.67 ± 0.09	60.27 ± 0.04	65.79 ± 0.09	4.37

Where $\bar{x}$ is the arithmetic mean, SD is the standard deviation, and °T is temperature.

**Table 7:** Particle size of starch after acid and enzymatic treatments with JM program.

Treatment	Variety			
	APE (μm)	YKW (μm)	HM (μm)	YA (μm)
	$\bar{x}$ ± SD *	$\bar{x}$ ± SD *	$\bar{x}$ ± SD *	$\bar{x}$ ± SD *
**Enzymatic**	36.84 ± 14.16 ^a^	27.52 ± 5.51 ^a^	37.43 ± 9.34 ^a^	33.01 ± 5.44 ^a^
**Acid**	37.96 ± 8.70 ^a^	31.96 ± 7.82 ^a^	30.77 ± 6.89 ^a^	34.68 ± 5.65 ^a^

Where $\bar{x}$ is the arithmetic mean; SD is the standard deviation; * shows significance; and different letters in a row indicate a significant difference, evaluated with Tukey’s test at 95% confidence.

**Table 8:** DLS particle size for ethanol-precipitated samples.

Treatment	Variety	Peak 1		Peak 2		Peak 3	
		(nm)	%	(nm)	%	(nm)	%
**Ethanolic** **Precipitation**	APE	14.6	2.5	91.8	21.7	476.3	75.8
	YKW	11.1	4.5	75.4	18.4	606.8	77.1
	YA	10.9	92.0	41.1	7.2	152.3	0.8
	HM	23.1	81.8	106.2	5.4	858.8	12.8

Where APE is Aq’hu Pukucho, YKW is Yurakk Kkachun Wakkachi, YA is Yurac Anca, and HM is Huarmi Mallco.

## Data Availability

The original contributions presented in the study are included in the article, further inquiries can be directed to the corresponding authors.
